# Characterization of Soybean WRKY Gene Family and Identification of Soybean WRKY Genes that Promote Resistance to Soybean Cyst Nematode

**DOI:** 10.1038/s41598-017-18235-8

**Published:** 2017-12-19

**Authors:** Yan Yang, Yuan Zhou, Yingjun Chi, Baofang Fan, Zhixiang Chen

**Affiliations:** 1 Department of Horticulture, Zijingang Campus, 866 Yuhangtang Road, Zhejiang University, Hangzhou, 310058 China; 2 Department of Botany and Plant Pathology and Purdue Center for Plant Biology, 915 W. State Street, Purdue University, West Lafayette, IN 47907 USA

## Abstract

WRKY proteins are a superfamily of plant transcription factors with important roles in plants. WRKY proteins have been extensively analyzed in plant species including Arabidopsis and rice. Here we report characterization of soybean WRKY gene family and their functional analysis in resistance to soybean cyst nematode (SCN), the most important soybean pathogen. Through search of the soybean genome, we identified 174 genes encoding WRKY proteins that can be classified into seven groups as established in other plants. WRKY variants including a WRKY-related protein unique to legumes have also been identified. Expression analysis reveals both diverse expression patterns in different soybean tissues and preferential expression of specific WRKY groups in certain tissues. Furthermore, a large number of soybean WRKY genes were responsive to salicylic acid. To identify soybean WRKY genes that promote soybean resistance to SCN, we first screened soybean WRKY genes for enhancing SCN resistance when over-expressed in transgenic soybean hairy roots. To confirm the results, we transformed five WRKY genes into a SCN-susceptible soybean cultivar and generated transgenic soybean lines. Transgenic soybean lines overexpressing three WRKY transgenes displayed increased resistance to SCN. Thus, WRKY genes could be explored to develop new soybean cultivars with enhanced resistance to SCN.

## Introduction

WRKY transcription factors are a class of DNA-binding proteins primarily found in plants and algae, although individual WRKY proteins have been identified in the human protozoan parasite *Giardia lamblia* and slime mold *Dictyostelium discoideum* ^1^. WRKY transcription factors contain the highly conserved WRKY domain composed of a conserved WRKYGQK sequence and a zinc-finger motif. WRKY transcription factors recognize a (T/A)TGAC(T/A) cis-regulatory element, also known as a W-box, in the promoters of target genes^2^. Based on the number and amino acid sequences of the WRKY domains, WRKY proteins are classified into three major groups^3^. Group I WRKY proteins contain two WRKY protein domains, whereas both groups II and III each possess only one WRKY domain. Group III WRKY proteins have a C2HC zinc finger instead of the C2H2 motif found in group I and II WRKY factors. Group II WRKY proteins can be further classified into five subgroups (IIa to IIe)^2,4^. While there are only one or a few WRKY genes in most algae, even moss (*Physcomitrella patens*), the earliest land plant, possesses at least 30 WRKY genes^1,4^. Vascular plants usually contain more than 50 genes encoding WRKY transcription factors with 72 in the model plant Arabidopsis^1,4,5^. Therefore, the WRKY gene family has expanded greatly during the evolution of land plants.

Since the identification of the first WRKY transcription factor in 1994^6^, great effort has been made in characterizing the roles of WRKY transcription factors in a diverse array of biological processes in plants. Several studies have demonstrated a critical role of WRKY transcription factors in plant development including successful male gametogenesis^7^, tolerance to interploidy crosses^8^, and embryo development^9^. WRKY proteins also participate in the regulation of seed size^10^, seed coat color^11^ and developmentally programmed leaf senescence^12,13^. The most prominent role of WRKY transcription factor family is the regulation of plant defense and stress responses. WRKY transcription factors play an important role in plant responses to microbial pathogens, as well as insect herbivory. Plants contain two interconnected branches of immunity: pathogen-associated molecular pattern (PAMP) triggered immunity (PTI) and effector-triggered immunity (ETI)^14^. WRKY transcription factors are critical components of the pathways responsible for the activation of PTI and ETI^15,16^. WRKY proteins are known to regulate plant responses to a wide range of abiotic stress including cold, drought, flooding, heat, heavy metal toxicity, low humidity, osmotic, oxidative, salt and UV stresses^17^. WRKY transcription factors also function in plant hormone signaling. A large number of plant WRKY genes are responsive to different plant hormones including salicylic acid (SA), jasmonate, ethylene, abscisic acid and gibberellin. Arabidopsis WRKY57 mediates crosstalk between jasmonate and auxin signaling, whereas WRKY70 moderates signaling between the jasmonate and SA^18^. Arabidopsis WRKY23 negatively regulates auxin responses by positively activating expression of biosynthetic genes for flavonols, which function as polar auxin transport inhibitors^19^. WRKY proteins are also shown to regulate plant metabolism. The first identified WRKY protein SPF1 regulates expression of β-amylase involved in catabolism of starch into sugars^6^. Other WRKY transcription factors regulate phosphate acquisition^20^, biosynthesis of lignin^21,22^ and other secondary metabolites including pharmaceutically valuable metabolites^23^.

Although substantial progresses have been made in the studies of plant WRKY proteins, our understanding of this important family of transcription regulators is still limited. This is in part because most of the reported studies on WRKY proteins are carried out in Arabidopsis with relatively few in important crop plants with special or unique biological or agronomic traits. For example, many legumes are important crop plants capable of fixing atmospheric nitrogen through intimate symbiosis with microorganisms. Among cultivated legumes, soybean is particularly important as a predominant plant source of proteins, cooking oil and other health-benefitting nutrients. There is also increasing interest in the use of soybean as a source of biomass for biofuel production. The soybean genome has been completely sequenced. Soybean is an ancient polyploid with two genome duplications that occurred approximately 59 and 13 million years ago, followed by gene loss and diversification and chromosome rearrangements^24^. The well-annotated genome sequences from soybean and other legumes can greatly facilitate identification and characterization of important genes such WRKY genes for analysis of the molecular and genetic basis of important legume traits using genetic, molecular and genomic tools. The insights from these studies can be explored to develop new strategies of improving important soybean traits including disease resistance and stress tolerance.

Soybean cyst nematode (SCN; *Heterodera glycines*) is the most important pathogen of soybean^25^. Use of SCN resistant cultivars is the primary management practice for SCN. However, many resistant soybean cultivars are derived from a few germplasms and their wide use has led to SCN race shift and overcoming of SCN resistance. Although other sources of SCN resistance have been identified, they cannot be incorporated efficiently through conventional breeding due to undesirable traits. The great progress over the last two decades in our understanding of plant immune systems against microbial pathogens have resulted in isolation of genes that can be deployed directly in crop plants through genetic engineering, bypassing the time-consuming introgression of resistance genes from otherwise undesirable sources (e.g. wild species) through conventional breeding. However, our knowledge about the genes and mechanisms important for plant-SCN interactions is very limited. The first two quantitative trait loci (QTLs) associated with soybean SCN resistance have been cloned and encode proteins unlike typical R proteins, suggesting unique molecular nature of soybean SCN resistance^25,26^. Therefore, a better understanding of the molecular basis of soybean SCN resistance is needed to develop new and novel SCN control strategies. Despite the well-established roles of WRKY proteins in plant immune system, there has been no reported study on their roles in plant responses to cyst nematodes.

In this report, we described the characterization of soybean WRKY gene family and their functional analysis in resistance to SCN. From the sequenced soybean genome, we identified 174 genes encoding WRKY proteins and classified them into seven groups as established in other plants. WRKY variants including a WRKY-related protein unique to legumes have also been identified. Soybean WRKY genes displayed diverse expression patterns in different soybean tissues and some of the soybean WRKY genes were preferentially expressed in certain tissues. To identify soybean WRKY genes that promote SCN resistance, we first screened the gene family for those members responsive to SA. Some of those SA-responsive WRKY genes were able to enhance SCN resistance when overexpressed in transgenic soybean hairy roots. We also generated stable transgenic soybean lines for five WRKY genes that promote SCN resistance in transgenic hairy roots and discovered that transgenic soybean lines overexpressing three of the five WRKY transgenes displayed substantially enhanced resistance to SCN. These results suggest that plant WRKY genes are involved in defense responses to SCN and can be exploited to enhance soybean resistance to SCN.

## Results

### Identification of soybean WRKY protein family

**Figure 1:**
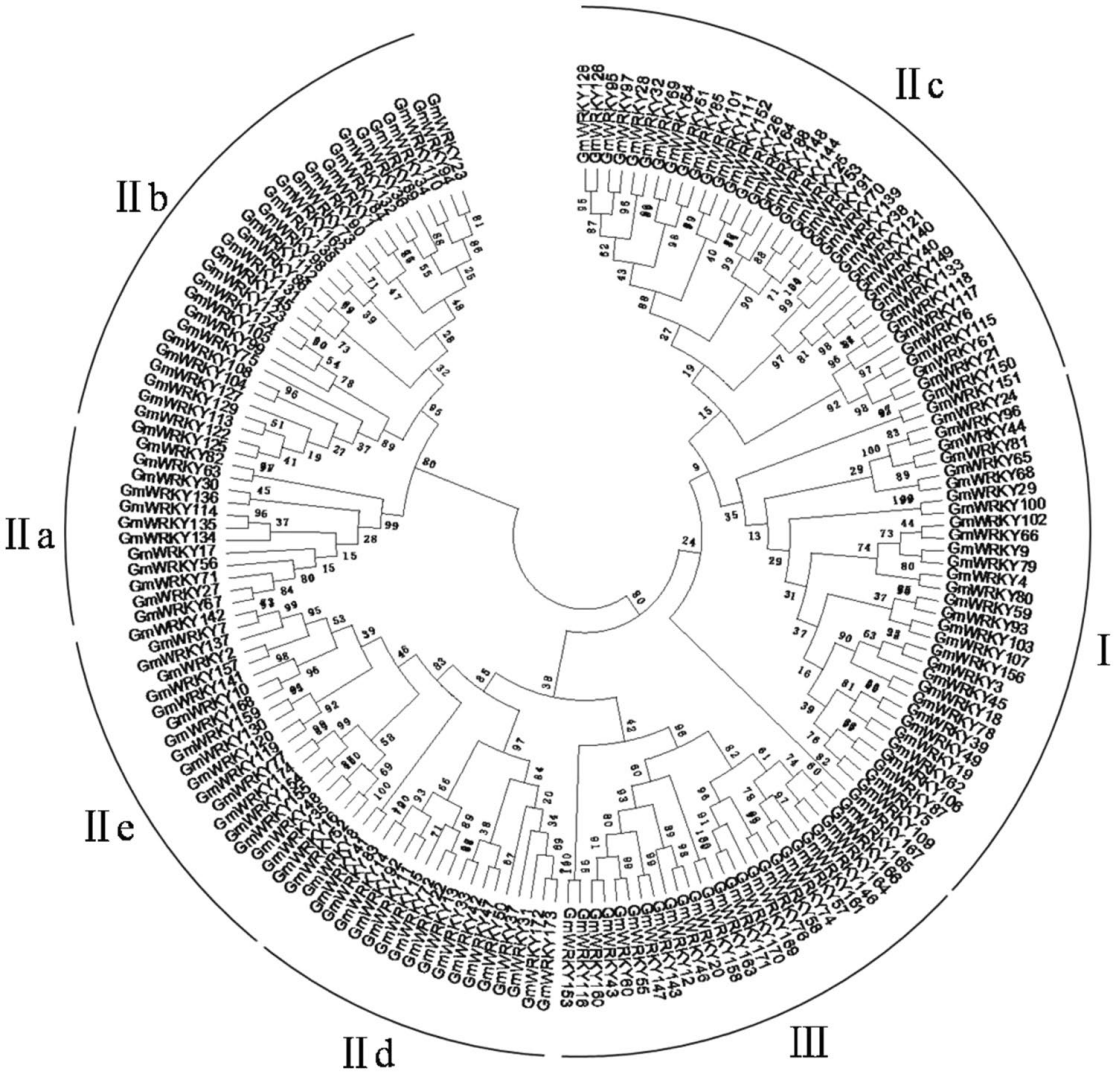
Phylogenetic analysis of soybean WRKY proteins. The conserved WRKY domain sequences of soybean WRKY proteins were used to construct multiple sequence alignments using Clustal W. Phylogenetic analysis was performed using MEGAv5.1. A phylogenetic tree was produced following neighbor-joining method using the aligned sequences. The phylogenetic tree was inferred using the the neighbor-joining method from the 174 soybean WRKY proteins identified. Bootstrap values from 1,000 replicates were used to assess the robustness of the tree. The soybean WRKY proteins are classified into seven well-defined groups (I, IIa to IIe and III), which are indicated.

**Figure 2:**
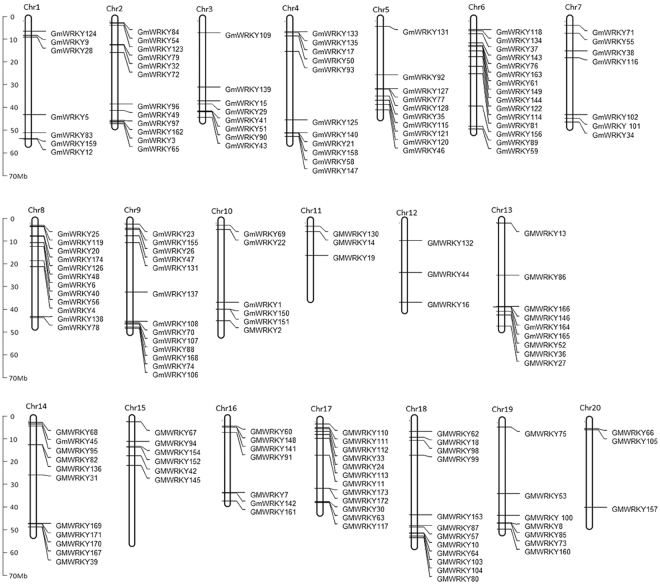
Distribution of soybean WRKY genes on soybean chromosomes.

Based on the search of the SMART database, it was reported in 2013 that the soybean WRKY gene family contains 133 members^27^. However, this reported soybean WRKY gene family is incomplete based on our more recent search. Based on the annotated genome of the soybean cultivar William 82 available in the Phytozome database (www.phytozome.net), we identified 174 genes encoding proteins containing the WRKY zinc finger motif in soybean (Fig. 1 and Supplemental Table 1). Thus, the WRKY gene family from soybean is substantially larger than those of other plants such as Arabidopsis (72 members)^28^ and rice (~100 members)^29^. The 174 WRKY genes are distributed on all 20 soybean chromosomes (Fig. 2). Most soybean chromosomes each contain more than 8 WRKY genes but chromosomes 11, 12 and 20 each contain only three WRKY genes (Fig. 2). Soybean is a paleopolyploid that has undergone at least two rounds of large-scale duplication approximately 13 and 59 million years ago^24^. Indeed, phylogenetic tree construction revealed that many soybean WRKY proteins have one or more close homologs (Fig. 1). Among the close soybean WRKY homologs, most are segmentally duplicated genes but a significant number of them are tandem repeats (*GmWRKY21* and 140 on Chromosome 4 and *GmWRKY7* and 142 on Chromosome 12). These observations suggest that a large number of close WRKY homologs in soybean resulted from duplication of chromosome regions or even whole chromosomes (polyploids), while local gene duplication also contributed to expansion of the WRKY gene family.

### Structural analysis of soybean WRKY proteins and WRKY variants

**Figure 3:**
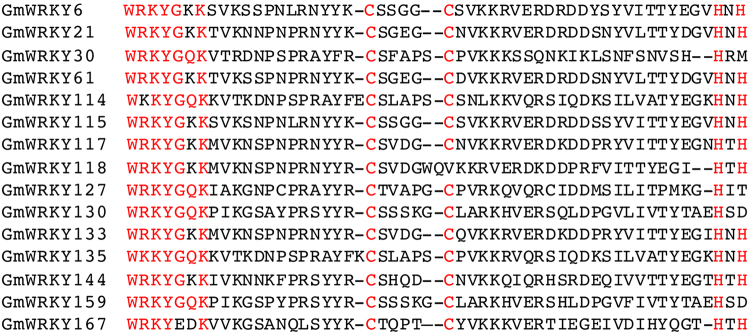
Identification of soybean WRKY variants. The conserved WRKYGQK residues and the coordinated zinc finger residues of cysteine and histidine are in red.

A WRKY domain contains the WRKYGQK signature sequence at the N-terminus and a zinc-finger motif (either Cx4-5Cx22-23HxH or Cx7Cx23HxC) at the C-terminus^2^. WRKY domains from a vast majority of soybean WRKY proteins contains these conserved sequences. Interestingly, at least 15 soybean WRKY proteins contain at least one amino acid substitution in either the WRKYGQK signature sequence or the C-terminal zinc-finger motif (Fig. 3). Among these soybean WRKY variants, five (GmWRKY30, 118, 127, 130 and 159) lack one of the four conserved zinc-finger residues (cysteine or histidine) and, therefore, are unlikely to form a zinc finger (Fig. 3). The remaining 10 WRKY variants contains one or two amino acid substitutions in their WRKYGQK signature sequence (Fig. 3). Among the 15 soybean WRKY protein variants is GmWRKY167, which contains a WRKY domain most closely related to the N-terminal WRKY domain of group I WRKY proteins (Fig. 3). However, GmWRKY167 does not contain the C-terminal WRKY domain, which is likely to be responsible for the sequence-specific DNA-binding activity of group I WRKY proteins^6,30^. In addition, the highly conserved WRKYGQK signature sequence of the WRKY domain was changed into WRKYEDK in GmWRKY167 (Fig. 3). We have recently reported that at its N-terminus, GmWRKY167 contains a Golgi-targeting transmembrane domain that is identified only in proteins from legumes^31,32^.

### Tissue- and Development-specific expression of soybean WRKY genes

**Figure 4:**
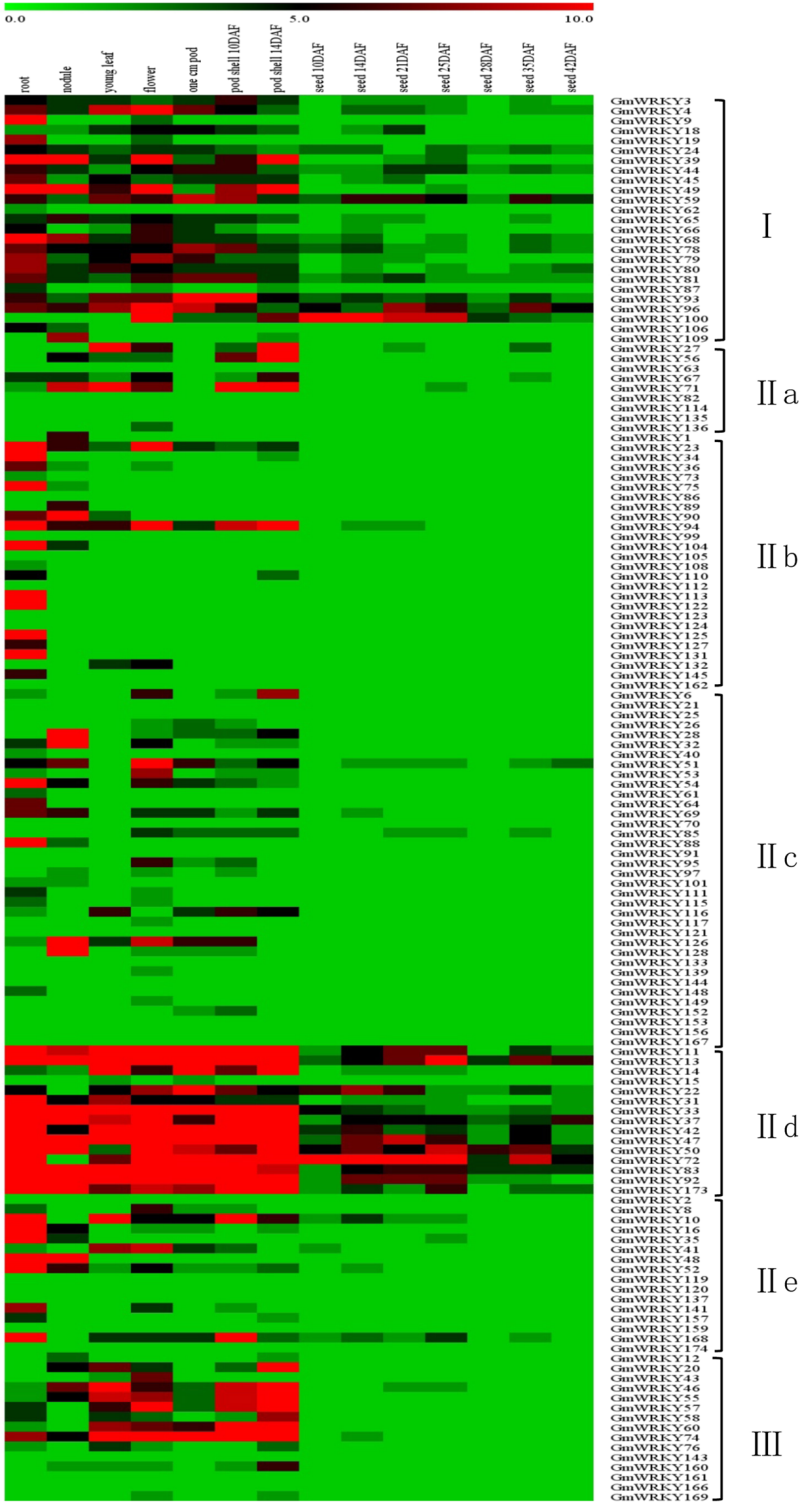
Tissue-specific expression profiles of soybean WRKY genes. Tissue- and development stage-specific expression of soybean WRKY genes. The expression data of soybean WRKY family in different tissues and development stages was downloaded from the RNA-seq database in soybase (http://soybase.org/soyseq/). Clustering and heat map analysis of the expression data was performed using MEV4.9.

In order to gain insights into the roles of WRKY genes in soybean growth and development, we downloaded the expression data of 142 soybean WRKY genes in 14 different tissues and developmental stages from the RNA-seq database in soybase (http://soybase.org/soyseq/) and analyzed them using MEV4.9 to form heat maps. As shown in Fig. 4, expression of a majority of soybean WRKY genes was detected in roots, nodules, young leaves, flowers or pods. On the other hand, expression of a relatively small number of soybean WRKY genes was detected at different stages of seed development (Fig. 4). For example, more than 20 of the 25 Group I WRKY genes analyzed were expressed at relatively high levels in at least one of the five tissues examined (roots, nodules, young leaves, flowers and pods) but only three of them (*GmWRKY59*, 96 and 100) were expressed at high levels in seeds (Fig. 4). Among the large number of Group II WRKY genes, most were expressed at low levels in both vegetative and reproductive tissues (Fig. 4). The Group IId WRKY genes, however, are the exception as almost all its members were expressed at high levels in roots, nodules, young leaves, flowers and pods (Fig. 4). A substantial number of Group IId WRKY genes were expressed at high levels at various stages of seed development (Fig. 4). A number of Group III WRKY genes were expressed at high levels in young leaves, flowers and pod but at low levels in seeds (Fig. 4). These results suggest possible roles of soybean WRKY genes in plant growth and development. It is also worth noting that some soybean WRKY genes including *GmWRKY2*8, 109 and 126 were expressed exclusively in nodules, suggesting a possible role in symbiosis with rhizobial bacteria (Fig. 4).

### Identification of SA-responsive soybean WRKY genes

**Figure 5:**
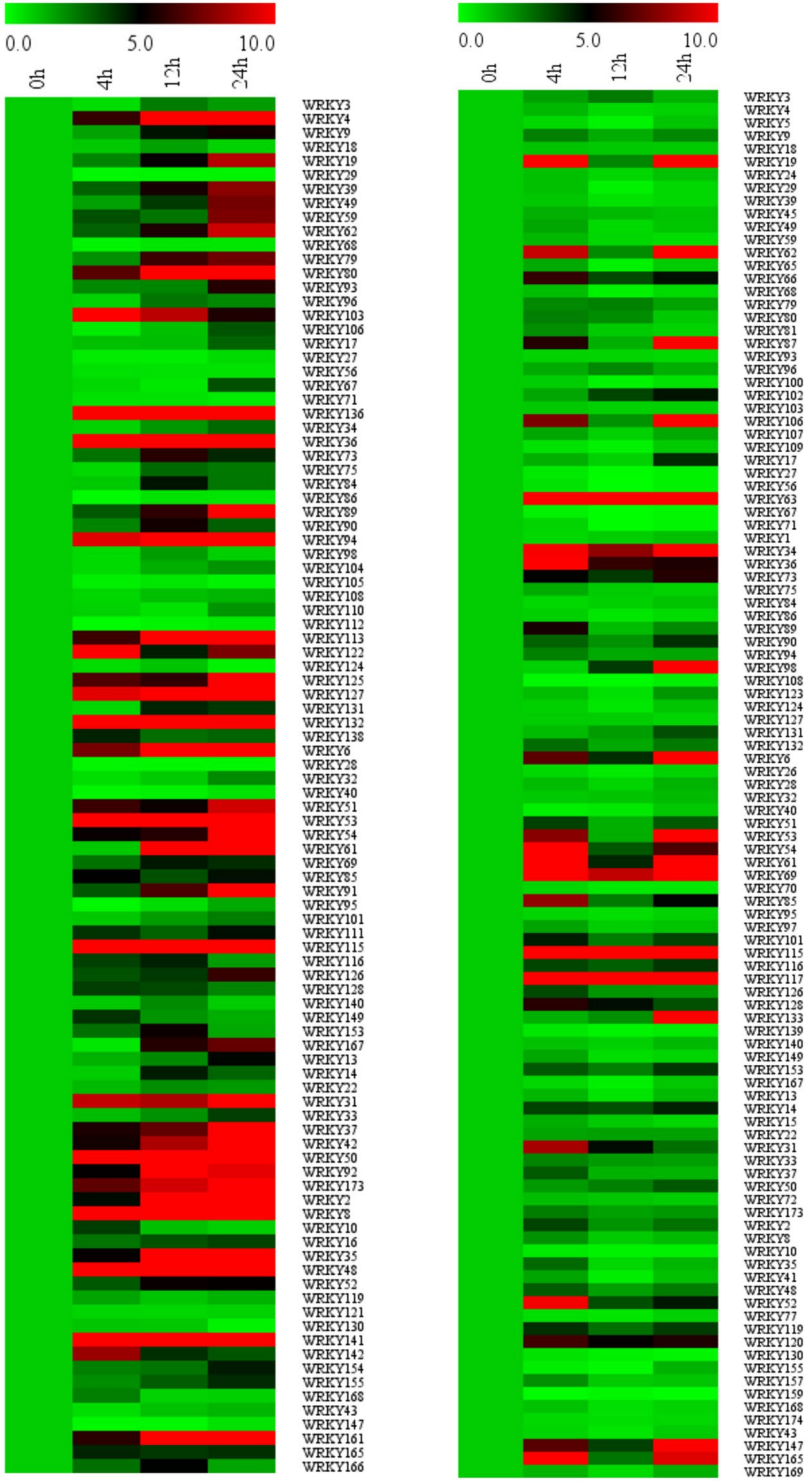
Expression of soybean WRKY genes to SA. Soybean WRKY gene expression in response to SA (1 mM) in leaves (**A**) and roots (**B**). Expression was determined by qRT-PCR.

Many plant WRKY and WRKY-interacting VQ genes are responsive to biotic and abiotic stresses and play important roles in plant stress tolerance and disease resistance^28,33^. To study the role of soybean WRKY genes in plant defense response, we analyzed their expression in response to SA, a signal molecule with a critical role in plant responses to a wide range of both biotic and abiotic stresses. Using qRT-PCR, we analyzed more than 100 soybean WRKY genes for their expression in both roots and leaves in response to SA (1 mM). In roots, more than 60% of soybean WRKY genes displayed an induction of more than 5-fold in expression in at least one of the three time points after SA treatment (Fig. 5). In leaves, approximately 40% of soybean WRKY genes displayed an induction of more than 5-fold in expression in at least one of the three time points after SA treatment (Fig. 5). Further analysis identified soybean WRKY genes whose expression was induced by SA primarily in roots (*GmWRKY4*,*39*, *49*, *59*, *79*, *80*, and 103), in leaves (*GmWRKY34* and 106), or in both roots and leaves (*GmWRKY19* and 62). Thus, a large percentage of soybean WRKY genes were responsive to SA.

### Effect of overexpression on resistance to SCN in transgenic soybean hairy roots

**Figure 6:**
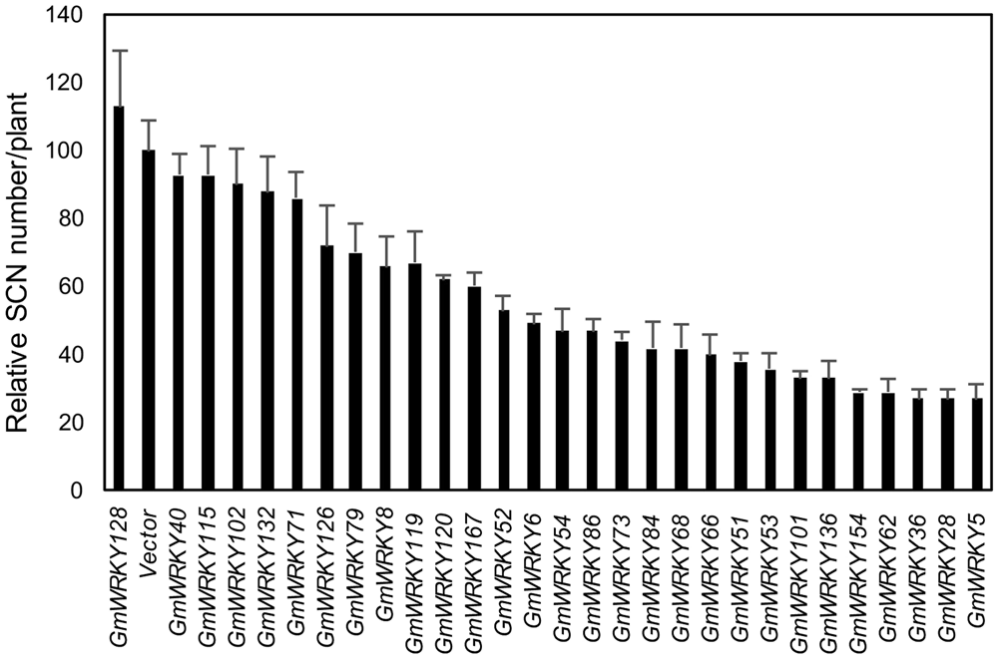
Assays of SCN resistance of transgenic hairy roots overexpressing soybean WRKY genes. Full-length WRKY coding sequences were cloned into the plant transformation vector pFGC5941 behind the CaMV *35 S* promoter. Composite soybean plants with transgenic hairy roots (in Williams 82) were generated using *A*. *rhizogene*s-mediated transformation. The cyst numbers per plants were counted 4 weeks after inoculation (20 eggs/cm^3^ soil) and are shown relative to those from control plants transformed with the empty pFGC5941 vector.

SCN is a devastating pest of soybean worldwide by damaging soybean root system, leading to stunting, wilting and yield loss for soybean plants. Deploying SCN resistant soybean cultivars is the primary means for fighting the billion-dollar disease. However, the narrow genetic basis of SCN-resistant soybean cultivars can lead to loss of resistance due to occurrence of new SCN races^34^. As plant WRKY proteins have important roles in plant defense against a wide range of biotic stresses, some members of the soybean WRKY gene family may also be involved in plant defense responses and can be exploited as regulatory genes to enhance plant disease resistance and stress tolerance through genetic engineering. We were particularly interested in identifying soybean WRKY genes whose expression can enhance soybean resistance to SCN because of the importance of the disease and the scarcity of genes known to promote soybean SCN resistance. Due to the large number of soybean genes, we designed a scheme of screening for soybean SCN resistance-promoting WRKY genes through first testing in transgenic hairy roots and then confirming in stable transgenic soybean plants. For this purpose, we chose approximately 30 soybean WRKY genes, many of which were highly responsive to SA, to test for their positive effect on resistance to SCN when overexpressed in soybean. We cloned the full-length coding sequences of the soybean WRKY genes behind the *CaMV 3*5* S* promoter in a plant transformation binary vector. These overexpression constructs were introduced into *Agrobacterium rhizogenes* and used to generate transgenic hairy roots in the SCN-susceptible cultivar Williams 82. Three weeks after transformation, composite soybean plants with transgenic roots were transplanted to pots. One weeks after transplanting, transgenic soybean roots were inoculated with a suspension of SCN eggs and SCN cyst numbers on each inoculated plant were counted after four weeks and compared to those in the transgenic roots containing the empty binary vector. The data in Fig. 6 showed the relative SCN number per plant calculated from four independent experiments with at least five replicates for each construct in each experiment. For the approximately 30 soybean WRKY genes tested, we observed a general trend of increased SCN resistance in the WRKY-overexpressing transgenic roots as indicated by the fact that all but one of these tested WRKY genes reduced the SCN number when overexpressed in transgenic roots (Fig. 6). Among these WRKY genes, about a half of them resulted in less than 50% reduction in the relative SCN number per plant, whereas the remaining half of these WRKY genes led to more than 50% reduction in the relative SCN number (Fig. 6). Importantly, five of these test WRKY genes (*GmWRKY1*5*4*, *62*, *36*, *28* and *5*) cause more than 70% reduction in the relative SCN number when overexpressed in transgenic roots (Fig. 6).

### Effect of overexpression on resistance to SCN in transgenic soybean plants

To confirm their effect on SCN resistance, we attempted to generate stable transgenic soybean plants for 11 soybean WRKY genes that were tested in transgenic hairy roots. Among the 11 soybean WRKY genes, five (*GmWRKY154*, *62*, *36*, *28* and *5*) were highly effective in promoting SCN resistance, causing more than 70% reduction in the SCN number when overexpressed in transgenic roots (Fig. 6). Four of the 11 soybean WRKY genes (*GmWRKY52*, *53*, *86* and 136) were modestly effective in promoting SCN resistance, causing approximately 40 to 60% reduction in the SCN number when overexpressed in transgenic roots (Fig. 6). *GmWRKY8* and 71, only the other hand, were ineffective in promoting SCN resistance, causing only 10 to 30% reduction in the SCN number when overexpressed in transgenic roots (Fig. 6). We attempted to generate transgenic overexpression lines for soybean WRKY genes that promoted soybean SCN resistance to various degrees when overexpressed in transgenic hairy roots to determine whether SCN resistance in transgenic hairy roots is a reliable indicator of SCN resistance in stable transgenic plants. We also performed sequence analysis to see whether these 11 soybean WRKY genes have functional homologs in Arabidopsis with a role in plant disease resistance. Sequence similarity was discovered between soybean GmWRKY5 and Arabidopsis AtWRKY33, which plays a critical role in plant resistance to necrotrophic pathogens and in tolerance to abiotic stresses^35–38^. In addition, GmWRKY136 was related to AtWRKY18, 40 and 60, three closely related Arabidopsis WRKY proteins with roles in plant immunity and stress tolerance^39–43^.

**Figure 7:**
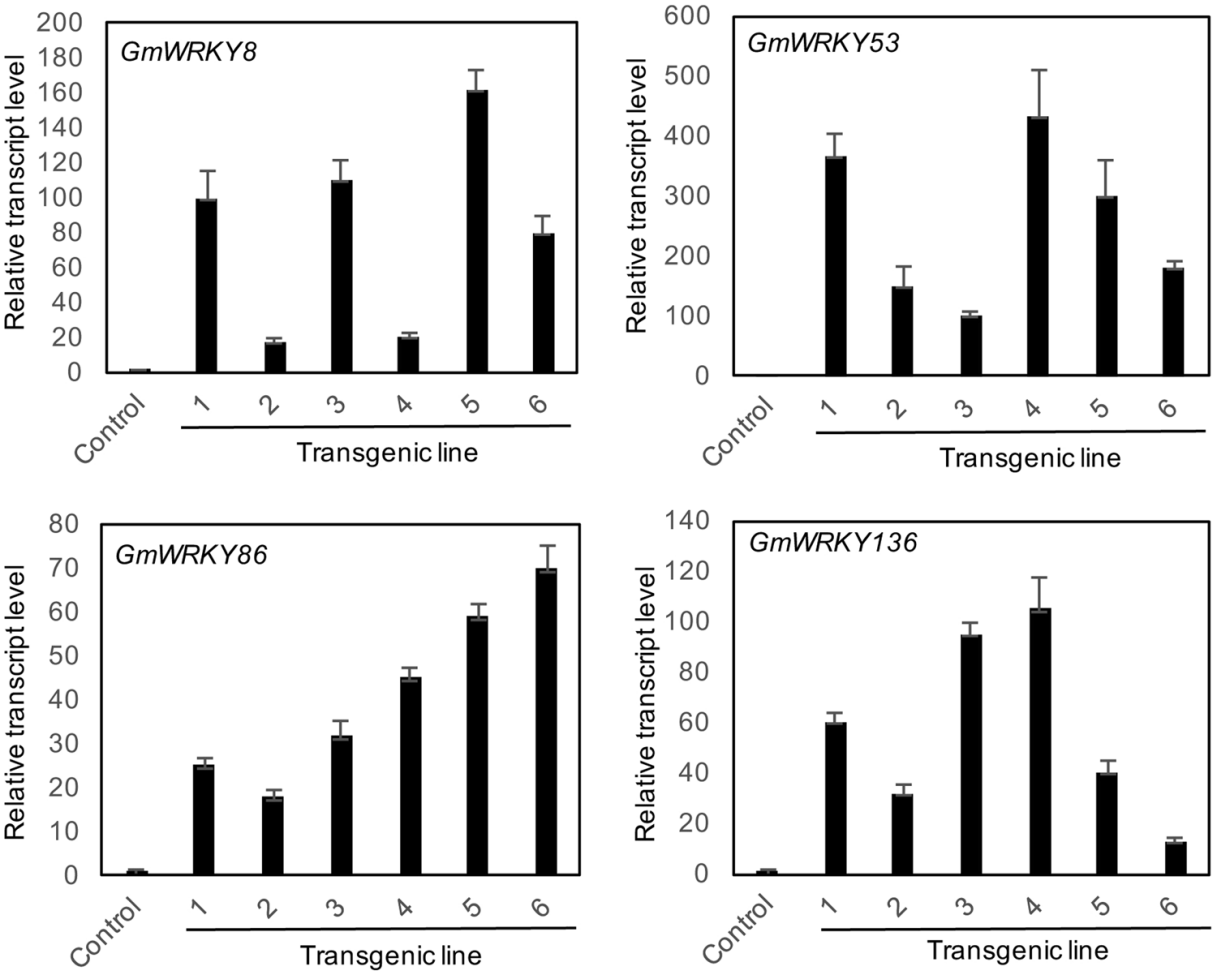
Overexpression of WRKY transgenes in transgenic soybean plants. Transgenic *GmWRKY soybean* plants (in Williams 82) were generated using *Agrobacterium*-mediated transformation. Expression levels of the transgenes in independent F2 transgenic lines were determined by qRT-PCR. Transgenic Williams 82 plants containing the empty pFGC5941 were used as control.

**Figure 8:**
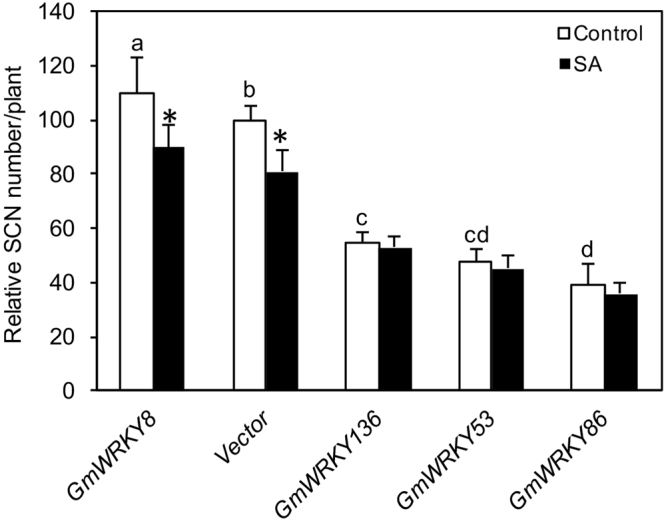
Assays of SCN resistance of transgenic soybean plants overexpressing soybean WRKY genes. Control and three independent lines with the highest levels of each transformed transgene were treated with H_2_O or 1 mM SA. The soybean seedlings were inoculated 24 hours after SA treatment with SCN and the nematode cyst numbers per plants were counted 4 weeks after inoculation. The cyst numbers are shown relative to those from control plants transformed with the empty pFGC5941 vector. According to Duncan’s multiple range test (*P* = 0.05), the mean cyst numbers per plant from untreated soybean plants do not differ significantly if they are indicated with the same letter. Stars denotes significant difference in the cyst numbers per plant in SA-treated plants compared with untreated plants of the same lines according to a *t* test (*P* = 0.05).

After multiple attempts, however, we were able to obtain stable transgenic lines for five soybean WRKY genes (*GmWRKY8*, *52*, *53*, *86* and 136). The reasons for the failure to obtain transgenic overexpression lines for other soybean WRKY genes are unclear. One possible reason could be the deleterious effects of overexpression of these WRKY genes on plant transformants as previously reported with other plant genes^18,44–46^. qRT-PCR analysis of T2 generation of these transgenic plants showed that none of the stable transgenic lines for *GmWRKY52* displayed increased expression of the transgene (data not shown). On the other hand, independent transgenic lines for the other four soybean WRKY transgenes (*GmWRKY8*, *53*, *86* and 136) with increased levels of transgene transcripts were obtained (Fig. 7). As expected from transgene expression driven by the *CaMV 35 S* promoter, the increase in the transcript levels for each transformed WRKY gene varied among different lines. For each transgene, at least three independent lines contain the transcript levels of the transformed transgene at least 40 times over those of control line (Fig. 7). For *GmWRKY8*, for example, we obtained three lines whose *GmWRKY8* transcript levels were increased by more than 100-fold relative to those in control plants (Fig. 7). For *GmWRKY*53, three lines contained more than 300 times more transcripts of the WRKY gene than those in control plants (Fig. 7). Despite the great increase in the expression of the WRKY genes, we observed no obvious changes in the growth or development of the transgenic plants (data not shown). To study the effect of the overexpression of the WRKY genes on soybean resistance to SCN, we inoculated the three highest overexpression lines for each WRKY transgene with SCN eggs and counted the cyst number per plant at four weeks post inoculation. As shown in Fig. 8, overexpression of *GmWRKY8* did not affect the SCN cyst number in transgenic soybean plants. On the other hand, transgenic soybean lines overexpressing *GmWRKY13*6, *53* and 86 had SCN cyst numbers 40–55% lower than those of control lines (Fig. 8). Thus, overexpression of these three soybean WRKY genes in transgenic soybean plants significantly increased soybean resistance to SCN. We also analyzed whether SA treatment could further increase SCN resistance of the transgenic lines. Control and transgenic soybean plants were treated with 1 mM SA at the time of SCN inoculation and the cyst numbers per plant were compared with those from untreated plants. As shown in Fig. 8, SA treatment significantly reduced the cyst numbers per plant of control and transgenic *GmWRKY8* plants but not the transgenic lines for the other three *GmWRKY* genes that already exhibited increased SCN resistance.

## Discussion

Soybean is an important crop that provides a large percentage of human’s dietary nitrogen. This is also increasing interest in the use of the legume crop as a source of biomass for biofuel production. Despite its importance, our understanding of many important soybean traits is still very limited. WRKY proteins are an important family of plant transcription factors with roles in a broad range of biological processes particularly in plant responses to biotic and abiotic stresses. Systematic identification and comprehensive characterization of WRKY proteins have been reported for a number of model plants including Arabidopsis and rice, which have provided important insights into the important family of plant transcription factors and associated biological processes. Identification and characterization of soybean WRKY proteins have also been reported^47–51^. With sequenced soybean genome, systematic identification and comprehensive analysis of the soybean WRKY gene family is now both possible and important. Through searching the sequenced genome of the soybean cultivar William 82 from the Phytozome database, we identified 174 WRKY genes. Thus, the WRKY gene family from soybean is substantially larger than those of other plants such as Arabidopsis (72 members) and rice (~100 members). As a paleopolyploid, soybean has undergone at least two rounds of large-scale duplication at approximately 13 and 59 million years ago. The large number of WRKY genes in soybean is most likely due to the polyploidy nature of soybean as revealed from the phylogenetic analysis, which revealed that many soybean WRKY proteins have one or more close homologs (Fig. 1). Among the close soybean WRKY homologs, most are segmentally duplicated genes, indicating that a large number of close WRKY homologs in soybean resulted from duplication of chromosome regions or even whole chromosomes.

Structural comparison of the WRKY domains of soybean WRKY proteins through both amino acid alignment and phylogenetic analysis revealed several interesting insights. All soybean WRKLY proteins contain WRKY domains that can be classified into the same groups previously identified in other plants (namely the N- and C-terminal WRKY domains of Group I and the single WRKY domain of Group IIa to IIe and Group III WRKY proteins) (Fig. 1). On the other hand, substitutions of amino acid residues in either the WRKYGQK signature sequence or the C-terminal zinc-finger motif occur in 15 soybean WRKY proteins (Fig. 3). Among these WRKY variants, five lacks one of the four conserved zinc-finger residues and therefore are unlikely to form a zinc finger and bind DNA (Fig. 3). The remaining 10 WRKY variants contains one or two amino acid substitutions in their WRKYGQK signature sequence (Fig. 3), which could alter or even abolish their DNA-binding activity. Soybean WRKY proteins with altered or abolished DNA-binding activity could lead to new molecular activities and novel biological functions. In addition, novel WRKY proteins that contain not only WRKY domains but also other structural domains such as kinase, RIR-LRR have also been identified^1^. In soybean, GmWRKY167 contains a WRKY domain and an N-terminal Golgi-targeting transmembrane domain that is identified only in proteins from legumes^31,32^. The highly conserved WRKYGQK signature sequence of GmWRKY167 was changed into WRKYEDK and the recombinant GmWRKY167 protein has no binding activity for W-box sequences^31,32^. Furthermore, silencing of *GmWRKY167* resulted in premature leaf senescence and reduction in nodule formation^31,32^. These findings support that structural modifications of duplicated soybean WRKY genes could lead to their functional diversification.

For functional characterization of the soybean WRKY gene family, we have analyzed their tissue-specific expression using the RNA-seq data. Soybean WRKY genes from a number of subfamilies including Group I, IId and III expressed at high levels in various tissues and organs (Fig. 4). In particular, a large percentage of Group IId WRKY genes expressed at high levels in all examined tissues including seeds, where expression of WRKY genes from other groups are generally very low (Fig. 4). The biological significance of the high basal levels of Group IId WRKY gene transcripts is unclear. Those Group IId WRKY genes with high expression levels could play roles in soybean growth and development. Alternatively, they may be involved in the regulation of basal levels of plant responses to biotic and abiotic stress. In addition, three WRKY genes (*GmWRKY26*, 106 and 126) were expressed exclusively in nodules (Fig. 4), suggesting a possible role in symbiosis with rhizobial bacteria. Using qRT-PCR, we also analyzed more than 100 soybean WRKY genes for their expression in both roots and leaves in response to SA and discovered a large number of SA-responsive soybean WRKY genes in roots, leaves or both (Fig. 5). Although SA-responsive WRKY genes have been identified in other plants including in Arabidopsis, most have been focused on those that are responsive to SA in shoots, particularly in leaves. Identification of SA-responsive WRKY genes in soybean roots could be significant in future studies of the roles of WRKY genes in plant responses to soil borne pathogens, either beneficial or pathogenic.

Resistant soybean varieties are the most economical means of managing SCN, the most important soybean pest. However, continued use of the same resistant variety can lead to change in the nematode population and breakdown of SCN resistance. In addition, highly levels of SCN resistance are often conferred by QTLs and their introgression into elite soybean cultivars can be time-consuming. In the present study, we have explored the large soybean WRKY gene family to identify specific WRKY genes that promote soybean resistance to SCN. Using first the composite soybean plants with transgenic hairy roots, we tested more than 30 soybean WRKY genes and identified WRKY genes that significantly increased SCN resistance when overexpressed in the roots (Fig. 6). We have also generated transgenic soybean plants and discovered that those transgenic lines overexpressing *GmWRKY13*6, 53 and 86 displayed significantly increased resistance to SCN (Figs 7 and 8). While these results are promising, they can be further improved through a number of approaches. First, we have only tested a limited number of soybean WRKY genes for possible effects on SCN resistance. In particular, we were able to generate stable transgenic soybean lines only for four soybean WRKY genes. By screening a large number of soybean WRKY genes through transgenic hairy roots and stable transgenic plants, it is likely that specific soybean WRKY genes with even stronger effects on soybean SCN resistance could be identified. Second, high levels of soybean resistance to SCN may require coordinated action of multiple genes, as evident from the QTLs underlying resistance to SCN in different soybean germplasms^52^. Therefore, once specific WRKY genes that confer moderate levels of SCN resistance are identified, they can be stacked either through genetic and molecular means to further increase the SCN resistance in transgenic overexpression lines.

In summary, we have performed a systematic search of the soybean genome and identified 174 soybean WRKY genes, making soybean among the plants with the largest WRKY gene family. Structural analysis identified structural WRKY proteins variants including a WRKY-related protein with important roles in regulation of leaf senescence and root nodulation^31,32^. Expression analysis revealed specific WRKY subfamilies with increased basal expression in different tissues or organs including developing seeds. Additional analysis also identified nodule-specific and SA-induced soybean WRKY genes that could play roles in soybean responses to biotic and abiotic stimuli. Through screens in transgenic hairy roots, we have identified a substantial number of soybean WRKY genes whose overexpression led to increased soybean resistance to SCN. The positive effects of soybean WRKY genes on resistance to SCN were confirmed in stable transgenic soybean plants. Thus, soybean WRKY genes should be explored as potential targets for genetic engineering to improve resistance to the most important soybean disease. It should be noted that we have also silenced the five genes in transgenic soybean hairy roots using artificial microRNAs but failed to observe significant effects on soybean SCN resistance. The lack of impact of silencing of the WRKY genes on SCN resistance despite the positive effect from their overexpression could result from functional redundancy of similar WRKY genes in soybean. It is also possible that some of these WRKY genes promote soybean SCN resistance only at high levels of expression but not at the physiological expression levels under their native promoters. Further analysis will be necessary to examine these possibilities to provide new insights into the biological roles of the WRKY genes in plant-nematode interactions.

## Methods

### Plant material and growth conditions

Soybean (*Glycine max* cv ‘Williams 82’) plants were grown in a greenhouse or a growth chamber at 25 °C with a 12/12 hour light/dark photoperiod.

### Phylogenetic analysis of soybean WRKY proteins

The conserved WRKY domain sequences of soybean WRKY proteins (see Supplementary Table S1) were used to construct multiple sequence alignments using Clustal W. Phylogenetic analysis was performed using MEGAv5.1. A phylogenetic tree was produced following neighbor-joining method using the aligned sequences.

### Tissue-specific expression profile analysis of soybean WRKY genes

The RNA-seq data of soybean WRKY genes in different tissues and development stages were downloaded from the soybase (http://soybase.org/soyseq/) and analyzed using MEV4.9 to form heat maps.

### qRT-PCR

Gene expression analysis using qRT-PCR was performed as previously described^53^. Soybean samples were lyophilized and stored at −80 °C until use. Total RNA was isolated from plant tissues using the Trizol reagent according to the supplier’s instruction. Extracted RNA was treated with DNase to remove contaminating DNA and reverse transcribed using the ReverTran Ace^®^ qPCR RT kit (Toyobo) for reverse transcriptase-PCR. qRT-PCR was performed with an StepOnePlus™ Real-Time PCR System (ABI). PCRs were performed using the SYBR^®^ Green qPCR Master Mixes (Takara) and gene-specific primers (Supplemental Table 2). Relative gene expression was calculated as previously described^54^. Soybean *Actin* gene (Glyma18g52780, 5′-GTGCACAATTGATGGACCAG-3′ and 5′-GCACCACCGGAGAGAAAATA-3′) was used as internal control.

### Generation of soybean with transgenic roots

For generating soybean with transgenic roots, the full-length coding sequences were amplified using gene specific primers (Supplemental Table 3). The amplified fragments were digested using appropriate restriction enzymes and inserted into the plant transformation vector pFGC5941 containing the CaMV 35S promoter^55^. Transgenic soybean hairy roots were generated as described previously^56^. The cotyledonary nodes of 3–5 days old soybean seedlings were infected by *Agrobacterium rhizogenes* K599 containing recombinant vectors. After cultivating at humidity condition for about two weeks, composite soybean plants with transgenic hair roots were transferred to soil.

### Generation of transgenic GmWRKYs soybean plants

For generating transgenic soybean plants, the overexpression binary vectors were transformed into Williams 82 wild-type plants using the cotyledonary node method^57,58^. Transgenic soybean plants in the T1 and T2 generations were confirmed with PCR using pFGC5941-specific primers and expression of the transgenes were analyzed by qRT-PCR with gene-specific primers.

### Method for assessing soybean SCN resistance in soybean plants

Eggs and second-stage juveniles (J2) were extracted from SCN cysts. Soybean seedlings were inoculated using a suspension of extracted eggs and J2 (20 eggs + J2/cm^3^ soil). Seedlings were incubated at 27 °C and 12 h day length. After 28 to 30 days, the cysts on the roots were counted. SCN resistance of the transgenic GmWRKY-overexpressing lines was determined based on comparison of their cyst numbers per plant with those from control plants transformed with the empty pFGC5941 vector.

## Electronic supplementary material

Supplemental Tables
