# Supplementary material for: Characterization of Soybean WRKY Gene Family and Identification of Soybean WRKY Genes that Promote Resistance to Soybean Cyst Nematode
Source: Sci Rep. 2017 Dec 19;7:17804. doi: 10.1038/s41598-017-18235-8 (PMC5736691; doi:10.1038/s41598-017-18235-8)
Supplement: Supplementary file 1 — Supplemental Tables [file 41598_2017_18235_MOESM1_ESM.pdf]

SUPPLEMENTARY INFOMRATION

Characterization of Soybean WRKY Gene Family and Identification of Soybean WRKY Genes that Promote Resistance to Soybean Cyst Nematode

Yan Yang, Yuan Zhou, Yingjun Chi, Baofang Fan and Zhixiang Chen

Supplemental Table 1. Soybean WRKY genes

Name	ID	WRKY group	Name	ID	WRKY group
GmWRKY1	Glyma.10G138300	IIb	*GmWRKY88*	Glyma09g254400	IIc
GmWRKY2	Glyma.10G230200	IIe	*GmWRKY89*	Glyma06g307700	IIb
GmWRKY3	Glyma.02G297400	I	*GmWRKY90*	Glyma.03G224700	IIb
GmWRKY4	Glyma.08G240800	I	*GmWRKY91*	Glyma.16G054400	IIc
GmWRKY5	Glyma.01G128100	I	*GmWRKY92*	Glyma.05G096500	IId
GmWRKY6	Glyma.08G142400	IIc	*GmWRKY93*	Glyma.04G115500	I
GmWRKY7	Glyma.16G176700	IIe	*GmWRKY94*	Glyma.15G110300	IIb
GmWRKY8	Glyma.19G217000	IIe	*GmWRKY95*	Glyma.14G028900	IIc
GmWRKY9	Glyma.01G053800	I	*GmWRKY96*	Glyma.02G203800	I
GmWRKY10	Glyma.18G238200	IIe	*GmWRKY97*	Glyma.02G285900	IIc
GmWRKY11	Glyma.17G168900	IId	*GmWRKY98*	Glyma.18G092200	IIb
GmWRKY12	Glyma.01G224800	III	*GmWRKY99*	Glyma.18G124700	IIb
GmWRKY13	Glyma.13G102000	IId	*GmWRKY100*	Glyma.19G177400	I
GmWRKY14	Glyma.11G053100	IId	*GmWRKY101*	Glyma.07G238000	IIc
GmWRKY15	Glyma.03G159700	IId	*GmWRKY102*	Glyma.07G227200	I
GmWRKY16	Glyma.12G212300	IIe	*GmWRKY103*	Glyma.18G242000	I
GmWRKY17	Glyma.04G061400	IIa	*GmWRKY104*	Glyma.18G256500	IIb
GmWRKY18	Glyma.18G081200	I	*GmWRKY105*	Glyma.20G030500	IIb
GmWRKY19	Glyma.11G163300	I	*GmWRKY106*	Glyma.09G280200	I
GmWRKY20	Glyma.08G021900	III	*GmWRKY107*	Glyma.09G250500	I
GmWRKY21	Glyma.04G218700	IIc	*GmWRKY108*	Glyma.09G240000	IIb
GmWRKY22	Glyma.10G032900	IId	*GmWRKY109*	Glyma.03G042700	I
GmWRKY23	Glyma.09G005700	IIb	*GmWRKY110*	Glyma.17G011400	IIb
GmWRKY24	Glyma.17G074000	I	*GmWRKY111*	Glyma.17G035400	IIc
GmWRKY25	Glyma.08G011300	IIc	*GmWRKY112*	Glyma.17G042300	IIb
GmWRKY26	Glyma.09G034300	IIc	*GmWRKY113*	Glyma.17G097900	IIb
GmWRKY27	Glyma.13G370100.	IIa	*GmWRKY114*	Glyma.06G212900	IIa
GmWRKY28	Glyma.01G056800	IIc	*GmWRKY115*	Glyma.05G184500	IIc
GmWRKY29	Glyma.03G176600	I	*GmWRKY116*	Glyma.07G133700	IIc
GmWRKY30	Glyma.17G222300	IIa	*GmWRKY117*	Glyma.17G224800	IIc
GmWRKY31	Glyma.14G135400	IId	*GmWRKY118*	Glyma.06G054500	IIc
GmWRKY32	Glyma.02G115200	IIc	*GmWRKY119*	Glyma.08G018300	IIe
GmWRKY33	Glyma.17G057100	IId	*GmWRKY120*	Glyma.05G211900	IIe
GmWRKY34	Glyma.07G262700	IIb	*GmWRKY121*	Glyma.05G185400	IIc
GmWRKY35	Glyma.05G160800	IIe	*GmWRKY122*	Glyma.06G190800	IIb
GmWRKY36	Glyma.13G310100	IIb	*GmWRKY123*	Glyma.02G020300	IIb
GmWRKY37	Glyma.06G077400	IId	*GmWRKY124*	Glyma.01G043300	IIb
GmWRKY38	Glyma.07G116300	IIc	*GmWRKY125*	Glyma.04G173500	IIb
GmWRKY39	Glyma.14G200200	I	*GmWRKY126*	Glyma.08G082400	IIc
GmWRKY40	Glyma.08G143400	IIc	*GmWRKY127*	Glyma.05G123000	IIb
GmWRKY41	Glyma.03G220100	IIe	*GmWRKY128*	Glyma.05G127600	IIc
GmWRKY42	Glyma.15G168200	IId	*GmWRKY129*	Glyma.05G029000	IIb
GmWRKY43	Glyma.03G256700	III	*GmWRKY130*	Glyma.11G021200	IIe
GmWRKY44	Glyma.12G152600	I	*GmWRKY131*	Glyma.09G080000	IIb
GmWRKY45	Glyma.14G016200	I	*GmWRKY132*	Glyma.12G097100	IIb

GmWRKY46	Glyma.05G215900	III	*GmWRKY133*	Glyma.04G054200	IIc
GmWRKY47	Glyma.09G061900	IId	*GmWRKY134*	Glyma.06g061900	IIa
GmWRKY48	Glyma.08G118200	IIe	*GmWRKY135*	Glyma.04G061300	IIa
GmWRKY49	Glyma.02G232600	I	*GmWRKY136*	Glyma.14G103100	IIa
GmWRKY50	Glyma.04G076200	IId	*GmWRKY137*	Glyma.09G129100	IIe
GmWRKY51	Glyma.03G220800	IIc	*GmWRKY138*	Glyma.08G320200	IIb
GmWRKY52	Glyma.13G289400	IIe	*GmWRKY139*	Glyma.03G109100	IIc
GmWRKY53	Glyma.19G094100	IIc	*GmWRKY140*	Glyma.04G218400	IIc
GmWRKY54	Glyma.02G010900	IIc	*GmWRKY141*	Glyma.16G031900	IIe
GmWRKY55	Glyma.07G057400	III	*GmWRKY142*	Glyma.16G177000	IIe
GmWRKY56	Glyma.08G218600	IIa	*GmWRKY143*	Glyma.06G125600	III
GmWRKY57	Glyma.18G213200	III	*GmWRKY144*	Glyma.06G168400	IIc
GmWRKY58	Glyma.04G223300	III	*GmWRKY145*	Glyma.15G186300	IIb
GmWRKY59	Glyma.06G320700	I	*GmWRKY146*	Glyma.13G267500	III
GmWRKY60	Glyma.16G026400	III	*GmWRKY147*	Glyma.04G238300	III
GmWRKY61	Glyma.06G147100	IIc	*GmWRKY148*	Glyma.16G031400	IIc
GmWRKY62	Glyma.18G056600	I	*GmWRKY149*	Glyma.06G147500	IIc
GmWRKY63	Glyma.17G222500	IIa	*GmWRKY150*	Glyma.10G171000	IIc
GmWRKY64	Glyma.18G238600	IIc	*GmWRKY151*	Glyma.10G171200	IIc
GmWRKY65	Glyma.02G306300	I	*GmWRKY152*	Glyma.15G139000	IIc
GmWRKY66	Glyma.20G028000	I	*GmWRKY153*	Glyma.18G183100	IIc
GmWRKY67	Glyma.15G003300	IIa	*GmWRKY154*	Glyma.15G135600	IIe
GmWRKY68	Glyma.14G006800	I	*GmWRKY155*	Glyma.09G029800	IIe
GmWRKY69	Glyma.10G011300	IIc	*GmWRKY156*	Glyma.06G242200	IIc
GmWRKY70	Glyma.09G244000	IIc	*GmWRKY157*	Glyma.20G163200	IIe
GmWRKY71	Glyma.07G023300	IIa	*GmWRKY158*	Glyma.04G223200	III
GmWRKY72	Glyma.02G141000	IId	*GmWRKY159*	Glyma.01G222300	IIe
GmWRKY73	Glyma.19G221700	IIb	*GmWRKY160*	Glyma.19G254800	III
GmWRKY74	Glyma.09G274000	III	*GmWRKY161*	Glyma.16G219800	III
GmWRKY75	Glyma.19G020600	IIb	*GmWRKY162*	Glyma.02G293400	IIb
GmWRKY76	Glyma.06G142000	III	*GmWRKY163*	Glyma.06G142100	III
GmWRKY77	Glyma.05G123600	IIe	*GmWRKY164*	Glyma.13G267600	III
GmWRKY78	Glyma.08G325800	I	*GmWRKY165*	Glyma.13G267700	III
GmWRKY79	Glyma.02G112100	I	*GmWRKY166*	Glyma.13G267400	III
GmWRKY80	Glyma.18G263400	I	*GmWRKY167*	Glyma.14G199800	IIc
GmWRKY81	Glyma.06G219800	I	*GmWRKY168*	Glyma.09G254800	IIe
GmWRKY82	Glyma.14G102900	IIa	*GmWRKY169*	Glyma.14G185800	III
GmWRKY83	Glyma.01G189100	IId	*GmWRKY170*	Glyma.14G186100	III
GmWRKY84	Glyma.02G007500	IIb	*GmWRKY171*	Glyma.14G186000	III
GmWRKY85	Glyma.19G217800	IIc	*GmWRKY172*	Glyma.17G197500	IId
GmWRKY86	Glyma.13G117600	IIb	*GmWRKY173*	Glyma.17G197500	IId
GmWRKY87	Glyma.18G208800	I	*GmWRKY174*	Glyma.08G078700	IIe

Supplemental Table 2. Primers for quantitative qRT-PCR

Gene name	Forward	Reverse
GmWRKY1	GCTCATCCAAGAGTCCCAAG	GACTATGGCTGAGGGTGGAA
GmWRKY2	GGCGAAAATACGGACAAAAA	GGTGGCGATGAAGAAGAAGA
GmWRKY3	TGGGTTTGTGTCAAACAGGA	TCTCCACATGTTTTCGGACA
GmWRKY4	AACTCACAAGGTTGGGTTGG	GCACGATGATTTTGGGTTCT
GmWRKY5	ATCCACTCAGGGCTTCTCAA	GGCTTGGGATGATTGTGACT
GmWRKY6	ACTCTCCTCCTTATCCCAATAATTAT	ACTGAATCCGTGAGTGGCATCATTG
GmWRKY8	ACCCCAACAACCAAAAGAATTATCCG	CTTCTTGTTCTATAATGATGTCTTGGCC
GmWRKY9	CTAACAAGCGCTCAAAAGACACCATG	AGATGATCAGCTGTTGCCTGACTTGA
GmWRKY10	GCAGTTTCTTCTTCTTCTTCTTCCTATTCT	TTTGGGTGGCGCAGAGG
GmWRKY13	CGCAAAACCTGTTATGCTTAAGTCA	AGCGATGATGGCGGGGCCG
GmWRKY14	CTGCGGAACCCAGAGTCTAC	CGAAGAGGACGAAAGTGGAG
GmWRKY15	GGTTTGGGTCATGCAAGAGT	TCATTTGTTGCTGCTGAAGG
GmWRKY16	AGCCGTCTTGCCGATAGAG	CTACGTCATCGAACCAGCCGAAGT
GmWRKY17	AGAAAGGCCGAGAGTGTGAA	AGCTTGGGGCATATGAACAC
GmWRKY18	AGTATGGGCAGAAGGTGGTG	TTTTCAGCAGCTGGGCTAAT
GmWRKY19	AGAGGAACTCATTGTCTGCCTCATC	ATCAAACTCATCTCCTCCTGATTTAG
GmWRKY22	AGTATTCTGGTGTTCCTTGG	CCTCACCACAGTTTCACCG
GmWRKY24	AGACCGAGTCGCCGAATCGCCTTCCGC	GCACCCGAATTAAGCGCGGTAGAGC
GmWRKY26	TCCCAACAACAACAACTCCA	ATTGACCGTAGCCTCATTCG
GmWRKY27	GAATCCGGATAAGGAGCACA	AGCTTGGAGCAAAAGAGCAC
GmWRKY28	AGCCTCCTCCGATCCCACCGCAGAT	TCGGCTGGTCCGGTTCGAGGTTCCAC
GmWRKY29	GAAGCACAAAGGGTGGTCAT	TGAGGGACTGTTTGGTGTCA
GmWRKY31	ATGGAGCACTTGATCCGAAC	GCAGGAGAAGGAAACTGCAC
GmWRKY32	CGTGGAGGCCGGTTCAGTCCTCTCT	ACTCGGTATCTCAGGCGGTTTCTCG
GmWRKY33	TGCAAAACCTGTTATGGTAAAGTCA	TGGCGGGGCCGATCTTG
GmWRKY34	TTGACTTCTTCTCCGGTGCT	GCATCTCCTTCAGCTTTTGG
GmWRKY35	TTCCGGACCCGGTCGAAC	CACGTCAGCATGGTACGT
GmWRKY36	GACACCAATGAAACTTCGCATTCCCAT	ATTGTTATTAGCAGCTACCCCTTGATCC
GmWRKY37	CCAGTCTCTAACCCTCGATT	TGAGACTGGCGGAGGAGTGAGGAAGAG
GmWRKY39	CAGACCAATGCCAAACAATG	CTAGGCTCCTCCTTGGTCCT
GmWRKY40	CGATGAGCAGGACCAGATACCTACA	GGTCTTCTCTTGACTTTTGAGCAGT
GmWRKY41	CCAAAAAGCAAGTAGAGAGATGCAGAACG	GTAACTTCATCACTGGGCTTATTA
GmWRKY42	CTCAACCAGAAACCCAACAA	GCGGCGGAGGGAGGGAG
GmWRKY43	TGGCCAAAAGGATATTCTCG	CAAAGGTGAAAATCCCCTCA
GmWRKY45	AACTCCGGCGAACCCCGCTCCAGTTC	AGGAATCGTGATGCACGGCGACCTC
GmWRKY48	ACCCGGTCCAACCCGAAC	GCCCGTGGCCATAATGGG
GmWRKY49	CACCACCTTCTCTGCCTCTC	GAGGTCCTTGTTGGGTTTGA
GmWRKY50	CTTCGCAGTCACAGAGTCTA	AGGAGTGAGGAAGAGGGAGGATCCTAGT
GmWRKY51	TTTCCCTCTATCGTCACACGTGTCA	CATAGAAAACCTCACTGGACGCGG
GmWRKY52	CCTCTCCCAAAATCAAATTCCTCCGCG	TCACGTCATCGAACCACCCGAAGTG
GmWRKY53	GCTAGTGATGATCATGAGAAGAGAC	CTTTTTATTATTGTTACTATTTCCCG
GmWRKY54	ATAGTCCTCTGCCTGAGTTACCACAG	TTCTTCTTCATCATCATCTTCTTCTGC
GmWRKY56	GACCAGAGATAACCCTTAC	TGAACAAGGCACTGAACCAAGGG
GmWRKY58	CTTGGTCCTGACGCAAATGA	TCCTTGAAATCAGACCAAAA
GmWRKY59	GATTGATGATAATGTTGCCCTTACTG	ATGCTTTCTGTATTCAACCTCGGA
GmWRKY61	GAATCGTCGGAGAAAGCAAC	CCCTTTCCACCCTTTTCTTC
GmWRKY62	CCTGATGCCAAAAGATGGAG	TGGATTTGGATTTCCCTTGACCACT
GmWRKY63	ACACAACACAAGGGCATGAG	AAATGGGAGCCATGTAGCAC
GmWRKY65	GCACCACACAAATCCTCTTTAGCCAC	GGTTCTTTGCCTTAGTCAATGAT
GmWRKY66	GTGGACCCCACGGCACCCTCGCCCA	GGCTGATATAGAGAATGGATGTTCAG
GmWRKY67	CCAACCGTTCAGTATCATGTTCAGCG	CGGGACTTCTGGTGATTCAGTTTTGGG

GmWRKY68	TGTGGAAAGGGCCTCTTATG	GCCTGATCACACGAAACTCA
GmWRKY69	CTACTTATCCCAGTATCAGCAGCAG	TTGATTACTACAAAGCCGTCTCTCTTGA
GmWRKY70	GGTGGCGCACCCAATTATGAGCTTCAA	ATCCGATGGTTGCTGCAGCGGCGATT
GmWRKY71	GCACAATCATCCTCAGTTTTCTTCCCAG	TGTTGAAGTGCTGAGAGATGCGGTAC
GmWRKY72	CAACCATCACTTCAAAAATCTGCAG	CAGCCTTTGCTGTTGCTGT
GmWRKY73	TCGGTTTCGGATGAAAAGAC	GGTTACCCTTGGCCATTTTT
GmWRKY75	CCCTTGATGAAGTGGAGGAA	GCTGGGGGTGTTATCTCTGA
GmWRKY77	TGGTTCATCTCGATCCCAACCATCG	TGGCTTGCAATAAGGGATTCCAATATC
GmWRKY79	GCATATCCAAGCCGAACATT	CGCTCGACCTTTTTCTTGAC
GmWRKY80	AACAGCTCCCACTGAACCAC	TTCTTCACGACGCAATTCAG
GmWRKY81	AGCACTCTACCAATGTTTTGAATG	TGGTTTTACTGCCGTATCCAAATCAC
GmWRKY84	GCCCTAACATCCCTTCTTCC	ATGACTTCCGCCATTGCTAC
GmWRKY85	CCTCTGCGGTTAAGGAACCACCTGA	GCTAGGTCTACAGTTTTATTCTGTTC
GmWRKY86	AGCGCGATCAGAATCATCTT	AGTGGTTGAGCCAGAAAGGA
GmWRKY87	CCTCTCCAGTCTCCCCTTCT	TCTTAAGCGGTTCCTGCACT
GmWRKY89	GCTGCTAATAATGGTACTAGCACTGG	GGAACTTTATGAGCATGCCCTGAGGGAC
GmWRKY90	CGCCTTGCAAAACCTTCCT	CCAAGATTCAATAGTTTAGTCAATTGAG
GmWRKY91	CTATGTTGTTCCCTATTTCCAATTCC	TCACCACCACCTTCGCCTCCGTTGT
GmWRKY92	CCTCCTCTTCTAAAACCATTCATT	GAAAGAGGAAGTCGAAAGCGGAGGC
GmWRKY93	GATTGATGATAATGTTGGCCTTACTG	ACTCACATCGGTCAATTGGGCTCC
GmWRKY94	TCCTCTCCTCGGATTCCCC	TGTGAAGAGGTGTTTCATCG
GmWRKY95	CATCCAATGAAGCTGAAGCA	TACACCGCACTTCTGACTCG
GmWRKY96	GAACGAGTTGTGGAATCGCTGTCCGA	CTCCGATGCTGCACAGCAA
GmWRKY97	ACAACCACCATGATCAGCAA	GCTTCTTGGGGTTTTCATCA
GmWRKY98	GCAACCCTCTCAACTTCAGC	GAATTTCGGGTCTCCAGTGA
GmWRKY100	AGCAGAAAGGGTGGTCATTG	GTTTTGCCGTTGGCTTGTAT
GmWRKY101	CCTTTCTCCCCTGCTTTCTCTAGCA	GTGGTGTTTTGGGGCCAGTCGAAT
GmWRKY102	AGGCTGTGCCAGCCCACTTCAATGTG	CTTATCAGCATTCACAGAAGAAGACTG
GmWRKY103	ATTGGAAGGCTGATGGACAG	AACCACTCTTGGCTCTCGAA
GmWRKY104	CACCAACATGTCCAGTCAGG	GACATGGTTTGGTGGTGACA
GmWRKY105	CATGCTCCAATCTCCCTCAT	ATTTTGGCATGCAAGTGAAG
GmWRKY106	GAGCTTCAACTGGAGGAACG	AAATAACCAGACCCGGGAAC
GmWRKY107	AACCGCTCAAGCACTTGTCT	CTGTGAAGCCCTTTCCACAT
GmWRKY108	CCCCACTAGGCCAGTTATCA	TTCAAGGGATTGGATGAAGC
GmWRKY109	CCTGAATATCCATCTACTCAGGGCTTCTCAG	CGGATTTTCGCTTCCTTTCACTTGTTTC
GmWRKY110	CAAATGCATCTTGCAGCAGT	AGTGGATGGATCCGTCGTAG
GmWRKY111	ACAACAGTTTCGGGACCAAG	TTCTCCTCATTCCCCATCTG
GmWRKY112	GCAAGCATTTGAGGCTTCTT	GTGCTGCTGATGTTGTGCTT
GmWRKY113	CAACAATGAATGACGGATGC	GGGGTTGCTGAAGGTATTGA
GmWRKY115	GGACTTCTACTTTGGAAACCCTCA	GATCACAGAAGGTTGCTTTTTCTG
GmWRKY116	CCAATGAGGACCCAGACACT	CAAACTTTCTTGGGCCATGT
GmWRKY117	CAGGTGAGTTTGGAGGAGGA	TCCTTGGATTGGGACTGTTT
GmWRKY119	CTGAACACAGCCATCCACAC	ATCATCCGTGAAACCACCAT
GmWRKY120	GAGTGTGGTGAAGCAGGTGA	GGTTGTTGGAGGAGGAACAA
GmWRKY122	CAACCTGGCAAGGGAATAAA	AGTGGAAGAGGGTGGTTGTG
GmWRKY123	AGGGAAGGAAGAGGAACCAA	CAGCACATCTTTGCACCTGT
GmWRKY124	TGTGATACCCAAACGATGAATG	GAGGGAGATTGAAGCATGGA
GmWRKY125	GGGTTTGTGTTAGGGCAAGA	GAGCCGGATAATAGCATGGA
GmWRKY126	AACCCTTGCGACCCTTAACT	AGCTCCTTGGGTAAGGGCTA
GmWRKY127	GAGCGAGTGAAAGAGGATGAGGAGCA	CTTTCATTGTTTGTTCTACAAC
GmWRKY128	ACACTACTACTGCTACTACTACCAC	ATTCTGGTGATGGTGGCCGGTCTTG
GmWRKY130	GGCGTAAATATGGGCAGAAA	AAGCCACGGTCATCTAATGC
GmWRKY131	CACATTGCACCACTACTCTC	GGTGTATGGCGGTCTTGTG

GmWRKY132	ATCTGCTCCATTCCCAACAG	CTGCCAATGCTGAAGTGAAA
GmWRKY133	TGGAGGAAGTAGCACCCACT	TGATTGTGGATGCCTTCGTA
GmWRKY136	ATCACTTGTCTCAAACCAAAGAGAAGCC	GGTATCCCTTGTCAAAGAAGTAGCC
GmWRKY138	AGCTTCAGCTCCATTTCCAA	CAGCCATAAGAGCTGCACTG
GmWRKY139	CCTCAAGCCATCCATGAAAT	CAGCTTTGGGATCCAGTGTT
GmWRKY140	GAGCCAAGGTTTTGCTTCAA	AGAAGGGGTTTGCGAGTCTT
GmWRKY141	TCCGCCGCCACAACCACCACTAAC	ATGGCCTCCTTGTTCAGCAG
GmWRKY142	CGGGAAGCACTAGAACCAAA	ATAAGAAGCGTCGGACATGG
GmWRKY147	CCCCAATGCTTTCAAAAAGA	AGAAGAACCCACGTTGTTGG
GmWRKY149	TCCATCAGTGCAATAGCTCCTTCAC	CATGCCCATGAATTCACTCTCGATC
GmWRKY153	CTCTCATCGGCCAGAGTTTC	GCAAGTGGAGCCCTTCATAG
GmWRKY155	GTGTGAAGGAAGAGAATATGGAAGACAT	CACCAAAACCCTGTGTGAAC
GmWRKY155	AAGCAGCTGCAACTTTTGGT	TGAGATCTGCACTCCTGTGG
GmWRKY157	TTCAAGCGCTACACCAACTG	GGGCAGTTGAAATTCGTGTT
GmWRKY159	CAGCTCCAAAGGGTGCTTAG	CGAAGCCACGGTCATCTAAT
GmWRKY161	GGCCACCAATGGAGAAAGTA	TGGTTGGAGTAGGGAAGGTG
GmWRKY165	TCCAGAGACTCAGGGTACGG	ACAATGGAGGGTCCTCTTGA
GmWRKY166	TGAGGTTGTTTTGCCTGTCA	TTTGGACCTGTTTGGTTGCT
GmWRKY167	CTGTTACGCGTTGAGTTCCTCTTTC	GAGCTTTTAGCATGAAACTCTTGG
GmWRKY168	CTTCTTCTTCCTCTTCCGG	CTCTTGCGTTGAAACAGAC
GmWRKY169	GCGAGAACAGATCCCAAAAG	GTGCCATTGCATGTGTGAAT
GmWRKY173	CGTTCACAACACTAACGCTTCCACC	CTTCGAGAACTCGAGCTCGACGGAT
GmWRKY174	TAAGAGTTCTGGGGCATTGG	TGTGTTGTCCACCAAGCAAT
GmACTIN	GTGCACAATTGATGGACCAG	GCACCACCGGAGAGAAAATA

Supplemental Table 3. Primers for generating overexpression constructs

Gene name	Primers
GmWRKY5	Forward: AGCCTCGAGATGAGCTTCAACATGAGAAACA<br>Reverse: AGCGGATCCTTAAAAGTTCTTTGACAAAAAAGAC
GmWRKY6	Forward: AGCCCATGGACTTCTACTTTGGAAACTCTC<br>Reverse: AGCTCTAGATCATTTGAAAGTAGTATCAGAATGTACG
GmWRKY8	Forward: AGCCCATGGACAATAATACTTGTGCCTCCT<br>Reverse: AGCTCTAGATCAAGAAGGGGCAACGG
GmWRKY28	Forward: AGCCCATGGAAGACAAAGACAGAGCA<br>Reverse: AGCTCTAGATCATCTGATGCGCATTCC
GmWRKY36	Forward: AGCCTCGAGATGGCAAGAGGGGGTGG<br>Reverse: AGCTCTAGATTATTTGTTGTTGCTTGATGTAATA
GmWRKY40	Forward: AGCCTCGAGATGTCAACAACTTCCCATCC<br>Reverse: AGCTCTAGACTACCATAATAAGAAGCTATCAAGTTGAG
GmWRKY51	Forward: AGCCCATGGAGAATACTAAGATGATGGGGGT<br>Reverse: AGCTCTAGACTAATCTTCTTTCAACATATGTGAA
GmWRKY52	Forward: AGCCTCGAGATGCATCACCGTAGATTCAGC<br>Reverse: AGCTCTAGATTATCCTGTGATGCCGCC
GmWRKY53	Forward: AGCCCATGGAGAATTATTCCATGTTGTTC<br>Reverse: AGCTCTAGATCAGAAGGGAGTGTATATTTGCAT
GmWRKY54	Forward: AGCCTCGAGATGGAGAAGAAGGAGATGGCT<br>Reverse: AGCACTAGTCTACTCTTCTTTCAACATGTGTGAA
GmWRKY62	Forward: AGCCTCGAGATGATCACTTCTTTCTCTGACCTTC<br>Reverse: AGCACTAGTTTACCTTGGATTTGGATTTCCC
GmWRKY66	Forward: AGCCTCGAGATGGTTGACGGCGGAAAA<br>Reverse: AGCTCTAGACTATGTGATCTGCTCTTCCTTCA
GmWRKY68	Forward: AGCCCATGGTTTCTACAGACAAAAGTGCG<br>Reverse: AGCTCTAGATTAGCTTTGGACCGGTTCAG
GmWRKY71	Forward: AGCCCATGGATTGTTCATCATGGATTA<br>Reverse: AGCTCTAGATTAATTATTGTGCAACAATCTTCCT
GmWRKY73	Forward: AGCCTCGAGATGGACAACCCACACCAC<br>Reverse: AGCTCTAGACTAGTTGGTGGAGAAAGTAGTGCAT
GmWRKY79	Forward: AGCCTCGAGATGGCTGGCGGCGGAGGT<br>Reverse: AGCTCTAGACTAGGTGATCTGCTCTTCCTTC
GmWRKY84	Forward: AGCCCATGGAAAACCATCACCAAATATTGCA<br>Reverse: AGCACTAGTCTAAGTGGATCCAGGCAACATAG
GmWRKY86	Forward: AGCCTCGAGATGGAGGGCAAAATTGCAT<br>Reverse: AGCTCTAGATTAATTTACTAATTTCTTGACAATTATTATC
GmWRKY101	Forward: AGCCCATGGCAAATTCTGTGGCATT<br>Reverse: AGCTCTAGACTAAACACGATCTTCACTCTCCT
GmWRKY102	Forward: AGCCTCGAGATGACACACCAGCAGATGCT<br>Reverse: AGCACTAGTTTATGTTATCTGCTCTTCCTTCAATC
GmWRKY115	Forward: AGCCCATGGACTTCTACTTTGGAAACC<br>Reverse: AGCTCTAGATCATTTGAAAGTAGTATCAGAATGTACGA
GmWRKY119	Forward: AGCCTCGAGATGGATGAATTTGCGTGCTT<br>Reverse: AGCTCTAGATTAAGGGTGTGGAAAATCATCCG

GmWRKY120	Forward: AGCCTCGAGATGGATGAATTTGCGTGCTT<br>Reverse: AGCACTAGTTAAGGGTGTGGAAAATCATCC
GmWRKY126	Forward: AGCCTCGAGATGTCTGTTGAACCTAAGGAACTT<br>Reverse: AGCTCTAGATTAATTCTGGTTATGATGGGACTT
GmWRKY128	Forward: AGCCTCGAGATGTCTGTTGAACCTAAGGAACT<br>Reverse: AGCTCTAGATTAATTCTGGTGATGGTGGCCGGT
GmWRKY132	Forward: AGCCTCGAGATGGATTCAGATCCAATTGG<br>Reverse: AGCTCTAGATTATTGCTTTCCGTTGCCATT
GmWRKY136	Forward: AGCCTCGAGATGGAATACGTGGATACTTCACTC<br>Reverse: AGCACTAGTTCACCATTGGGCCTCTAGAAT
GmWRKY154	Forward: AGCCCATGGCGGAGGAGAAAAGAGA<br>Reverse: AGCTCTAGATCAATCCTTCTCCTCCTTCCTC
GmWRKY167	Forward: AGCCTCGAGATGCCTTTCCGAAATTCCAA<br>Reverse: AGCTCTAGATCATATTTCATTTGGATATTGAGGAGTTCT
